# Atlanta Medical and Surgical Union

**Published:** 1884-11

**Authors:** 


### ATLANTA MEDICAL AND SURGICAL UNION.

Reported for THE ATLANTA MEDICAL AND SURGICAL JOURNAL.

STATED MEETING, October 7, 1884.

Dr. W. S. Elkin, president, in the chair.

The discussion was opened by Dr. A. S. Dyar by reading a paper on Septicæmia as follows:

There is perhaps no subject upon which the general practitioner should be better informed than that of septicæmia. It is one that the practitioner meets very often in the course of his practice, and I regret to say that I do not believe that the profession give as much attention to septicæmia as its importance demands. Being very rapid in its course and exceedingly fatal, we should try and prepare ourselves as thoroughly as possible to battle with this most dreaded of all affections of the blood.

Septicæmia, as we all know, is a poisoning of the blood—the absorption of some poisonous or putrid matter in the blood. The term itself denotes the presence in the blood of septic matter. There is also another affection of the blood known as pyæmia, considered by many very eminent surgeons and pathologists as analogous to septicæmia. Even the celebrated Virchow believed them to be one and the same, his views being supported by a great many who declared if there was a distinction, it was "a distinction without a difference."

However, I believe that there is a distinction, and that they are separate and distinct affections, as recent experiments and investigations have proved. Pyæmia, as the term indicates, is a pus in the blood—purulent matter. Not only do the symptoms in pyæmia and septicæmia differ somewhat, but there is a marked difference in the pathological characteristics of the two. From recent experiments by MM Coze & Fitz it has been clearly shown that while an abundance of bacteria are always found in the blood of a septicæmic patient, they are missing in the blood of pyæmic patients. We therefore see that pathological researches support us in our views of a difference between the two. We conclude, then, that a pyæmia may be a septicæmia, but a septicæmia is not always a pyæmia.

SYMPTOMS AND COURSE OF SEPTICÆMIA.

We usually find it ushered in by a marked rigor, followed by a profuse and exhausting diaphoresis. These rigors may afterwards occur at regular intervals (though not always) being discriminated from malarial rigors by the absence of the fever that immediately follows this class of rigor. The temperature is very high, often reaching 108° Fahr. We find, however, that under the influence of the profuse diaphoresis following the rigor, the temperature falls very rapidly. The pulse is rarely below 90, frequently reaching 130 to 180, and it is even claimed to go so high as 200. Respiration hurried, often being from 40 to 50 per minute, and it is claimed by some even more than this. There is unavoidably a jaundiced appearance of the patient, flushed countenance, with miliary eruption.

The patient very often assumes a soporific condition, from which it is very difficult to fully arouse him. Still, he seems to exhibit a semi-conscious condition, putting ou this tongue when told to do so, and taking medicine in a mechanical way, not realizing what he is doing.

This condition in my experience has proved very unfavorable. It is not an unfrequent occurrence to see the patient very delirious, particularly at night, the attendants often having to exert force to confine them to their beds. The tongue is usually furred and dry, before death becoming hard, brown and dry, sometimes cracked from which a little hemorrhage is seen; sordes accumulates upon the lips and gums, a condition of carphologia and subsultus tendinum, after which death is almost certain to ensue, the patient usually dying in a comatose condition.

The prognosis of septicæmia is always unfavorable, though we have a great many cases of the mild type that do recover. However, in these cases we find the amount of septic material introduced into the system to have been comparatively small. These mild cases may continue for weeks and months before the septic material is fully eliminated; but in the more acute cases death usually results in from three to fourteen days.

TREATMENT.

There is no specific for septicæmia. What, then, must we do? Treat it from a common-sense view. There are two cardinal points we must always observe in the treatment—one, the prophylactic, and the other the curative. Now, as to the so-called curative treatment. I do not belive that any remedy possesses a curative power in septicæmia, but that our prime object is to so stimulate the system that the patient will be enabled to bridge over the attack without succumbing to the poisonous element that fills his blood, trying to rob him of life. What, then, must we use? Every tonic and stimulant has been used and pushed to its farthest extent. But in my opinion (and I think statistics will bear me out in it) there is nothing so efficacious as quinia in large doses. Besides this, I give milk punch frequently in small quantity, allowing the patient to eat plenty of light, nutritious food in small quantities, but at short intervals. The quinia, besides possessing tonic properties, is said to be an antiseptic, which, if true, is just what we so much need. Socin, of Basle, during the late Franco-Prussian war, in cases of septicæmia, gave quinia in from 90 to 105 grs. in twenty-four hours, with splendid success. The bowels should be kept open by some mild purgative, and the physician should be careful to see that the urine is voided regularly, as in the latter soporific stages the patient, of course, pays no attention to this.

Now as to the prophylatic treatment. In this case as in the curative, every prophylactic remedy has been used, most prominent of which are bromine, carbolic acid, chlorine, boracic acid, antiseptic oils, permanganate of potash and others. All of which, no doubt, possess some antiseptic virtue.

But, first of all, we must look to the immediate surroundings, and see that everything is as far as possible conducive to the recovery of the patient. If the patient is in a crowded hospital or in any room where there is another or others sick, he should be removed at once to himself. I do not believe septicæmia to be infectious, but do believe it to be contagious. Therefore, it is prudent, as far as possible, to isolate septicæmic patients from others who may be in danger of contracting it.

When we have done this, we should then see to placing proper disinfectants in the room, and that any dressings in case of an open wound are properly and carefully disinfected. We can now advance still further as to the employment of our prophylactic treatment. Accepting the theory that septicæmia is dependent upon bacteria, whether these bacteria are the very incarnation of the poison or merely serve to transport it, is a question that is not as yet definitely settled, and consequently need not detain us here. That they do exist in septicæmia, however, is conclusively proved by the experiment of Charveau and Sanderson; that a septic fluid demonstrated to have produced toxemia when injected in the veins of a living animal be taken and strained through a porcelain filter, the liquor so filtered may then be injected with impunity, whereas the solid residue retains in full force all the septic matter of the original fluid. This residue is shown to consist entirely of bacteria. Assuming, then, that septicæmia is dependent upon septic material for development, and that septic material is caused by putrefying organic matter, and that putrefaction requires the presence of living germs, we then naturally conclude that to stop the progress of this affection we must use some remedy to destroy the life of these bacteria. We naturally question, then, to what must we resort? By reference to experiments recently performed we will see that it has been proved beyond doubt that ozone destroys bacterian life; therefore, we naturally conclude that in ozone we have found the long sought-for remedy to prevent the spread, progress, and possibly arrest the development of septicæmia.

Dr. Lancaster stated that he had recently seen a case of gunshot wound of the thigh, which seemed to be doing well for the first few days, after which time, the patient became paralyzed and had severe convulsions and died in a few days. He asked if the patient might not have suffered from septicæmia.

Dr. Gray said that he could see no evidence of blood-poisoning, but thought the patient died with tetanus.

The President said that he regretted to see so little interest taken in the discussion of the paper of Dr. Dyar and hoped that future, papers would be more generally discussed.

Dr. Noble presented and demonstrated the working of a new bladder douche. The douche consists of an ordinary fountain syringe with a catheter attached. Between the catheter and fountain is placed a two-way stop cock and waste-tube. The cock, opened as indicated by its being turned parallel with the supply tube, permits the influx of water to the bladder; when it is desired to relieve the bladder of this water the cock is turned parallel to the waste-pipe, when the water returns by the same catheter and tube as far as the cock and escapes by the waste or drain pipe. At the extreme upper end of this pipe is a small vent for the admission of air to prevent syphon action. The advantages claimed for this instrument are: Most perfect and easy mode of action, complete distention and perfect cleansing of the bladder, the large size of the calibre of catheter, its applicability in all cases where cavities need douching. It admits no air into the cavity when being douched.

